# Chromosome and Megaplasmid Sequences of *Borrelia anserina* (Sakharoff 1891), the Agent of Avian Spirochetosis and Type Species of the Genus

**DOI:** 10.1128/genomeA.00018-17

**Published:** 2017-03-16

**Authors:** Haitham Elbir, Parth Sitlani, Sven Bergström, Alan G. Barbour

**Affiliations:** a Department of Molecular Biology and the Laboratory of Molecular Infection Medicine, Umeå University, Umeå, Sweden; b Departments of Microbiology and Molecular Genetics and Medicine, University of California Irvine, Irvine, California, USA

## Abstract

Sequences of the linear chromosome and plasmids of *Borrelia anserina*, the cause of avian spirochetosis of poultry, revealed a smaller genome than those of other *Borrelia* spp. transmitted by argasid ticks. Missing or disrupted genes included a *dam* methylase and those in the pathway for synthesis of phospholipids from glycerol.

## GENOME ANNOUNCEMENT

*Borrelia anserina* is the globally distributed agent of avian spirochetosis, a tick-transmitted disease of poultry (1). *B. anserina* is phenotypically distinguished from other species in the relapsing fever group by a host range limited to birds and the exclusive use of *Argas* sp. soft ticks as vectors. *B. anserina* has a linear chromosome of ~900 kb and a megaplasmid, like other members of the genus, but fewer plasmids in total (2, 3).

*B. anserina* strain Es (ATCC 49835) had been isolated from a domestic chicken in California (4) and was cultivated in Barbour-Stoenner-Kelly medium. Genomic DNA was extracted with phenol-chloroform after lysis in sodium dodecyl sulfate and proteinase K. For sequencing, the single-molecule, real-time long-read approach on a Pacific Biosciences (PacBio) RS I instrument (Menlo Park, CA, USA) was combined with error-correction with short single reads from an Ion Torrent apparatus (Life Technologies, Inc., Carlsbad, CA, USA), as previously described (5, 6).

The 56,438 PacBio reads (*N*_50_, 20,171 nucleotides [nt]) provide chromosome and megaplasmid coverages of 662× and 410×, respectively. These were assembled with the Hierarchical Genome Assembly Process 2 (PacBio). The 2,394,657 Ion Torrent single reads had a mean length of 148 nt, and the chromosome and megaplasmid coverages were 227× and 340×, respectively. The Assembly Cell of Genomics Workbench version 8.5 (Qiagen, Denmark) was used for short reads. Gene prediction was completed with the Prokaryotic Genome Automatic Annotation Pipeline (http://www.ncbi.nlm.nih.gov/genome/annotation_prok), followed by manual annotation.

The linear chromosome comprised 906,833 bp, with a G+C content of 29.5%, and 799 protein-coding sequences, 32 tRNAs, three rRNAs (5S, 16S, and 23S), and seven pseudogenes. Gene order was generally syntenic with that of *B. hermsii* (CP00048). The maximum cumulative GC skew was at position ~453,000. The sequence length was consistent with the smaller size of the *B. anserina* chromosome by pulsed-field gel electrophoresis (3). Alignment of the strain Es sequence with the 904,790-nt sequence of strain BA2’s chromosome (CP005829) identified four transversions and 37 single-nucleotide indels.

The unique absence of *dam* methylation of *B. anserina* DNA, as previously reported (7), was confirmed by base analysis with PacBio’s SMRT Analysis for 6-methyladenine modification (8). Locus N187_02280 is orthologous to a methylase-coding sequence of *B. hermsii* but is a pseudogene with multiple frameshifts.

*B. anserina* has a *glpQ* gene (9) but lacks *glpA*, *glpF*, and *glpK*, and *glpT* is partial. Thus, it can acquire glycerol-3-phosphate for phospholipid synthesis from dihydroxyacetone phosphate, but, unlike other *Borreliaceae* spp., not from environmental or salvaged glycerol (9).

Megaplasmid lpA89’s length of 89,872 bp (G+C content, 28.8%) was consistent with reported pulsed-field gel electrophoretic migrations (2, 3). The shorter length of lpA89, which otherwise was largely collinear with *B. hermsii*’s 183-kb megaplasmid (CP0143450), was accounted for by gene loss (e.g., for factor H-binding protein and chitobiose transport proteins) and by fewer paralogs in the gene families of megaplasmids (10).

### Accession number(s).

Sequences for the chromosome and megaplasmid have been deposited in the GenBank/DDBJ/EMBL database under accession numbers CP013704 and CP014325 (BioProject PRJNA311246 and BioSample SAMN04481062). Associated sequences are complete plasmids pB25 (CP014520) and cp5 (CP014521) and a plasmid fragment (CP018882).
